# Supplementary material for: Luminal STIM1 Mutants that Cause Tubular Aggregate Myopathy Promote Autophagic Processes
Source: Int J Mol Sci. 2020 Jun 21;21(12):4410. doi: 10.3390/ijms21124410 (PMC7352373; doi:10.3390/ijms21124410)
Supplement: Supplementary file 1 [file ijms-21-04410-s001.pdf]

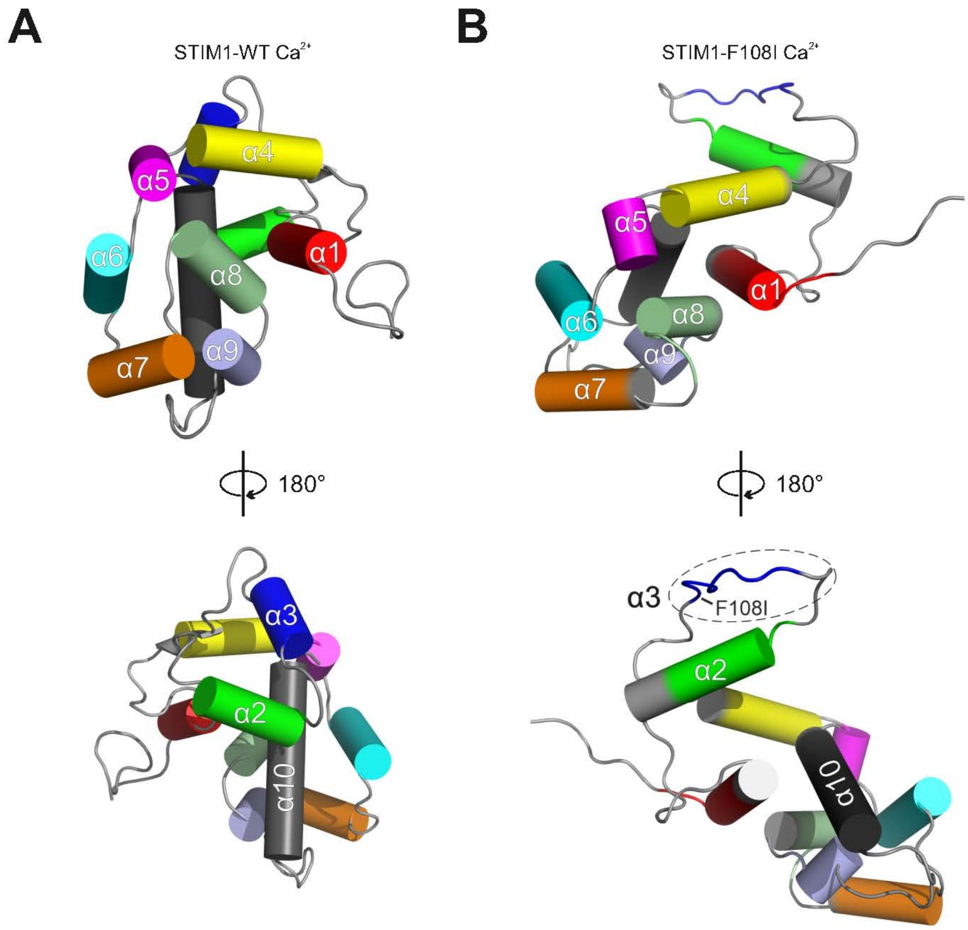

Supplementary Figure 1: Conformational rearrangement of the luminal STIM1 F108I mutant. (a,b) Two orientations are shown for the Ca^{2+}-bound resting state of STIM1 based on NMR results [9] and for STIM1-F108I. The helices (shown as cylinders) are labeled as follows: canonical EF hand (helix 1 and 2), non-canonical EF hand (helix 3 and 4), a short connecting helix 5 and the SAM domain (helix 6 to helix 10).

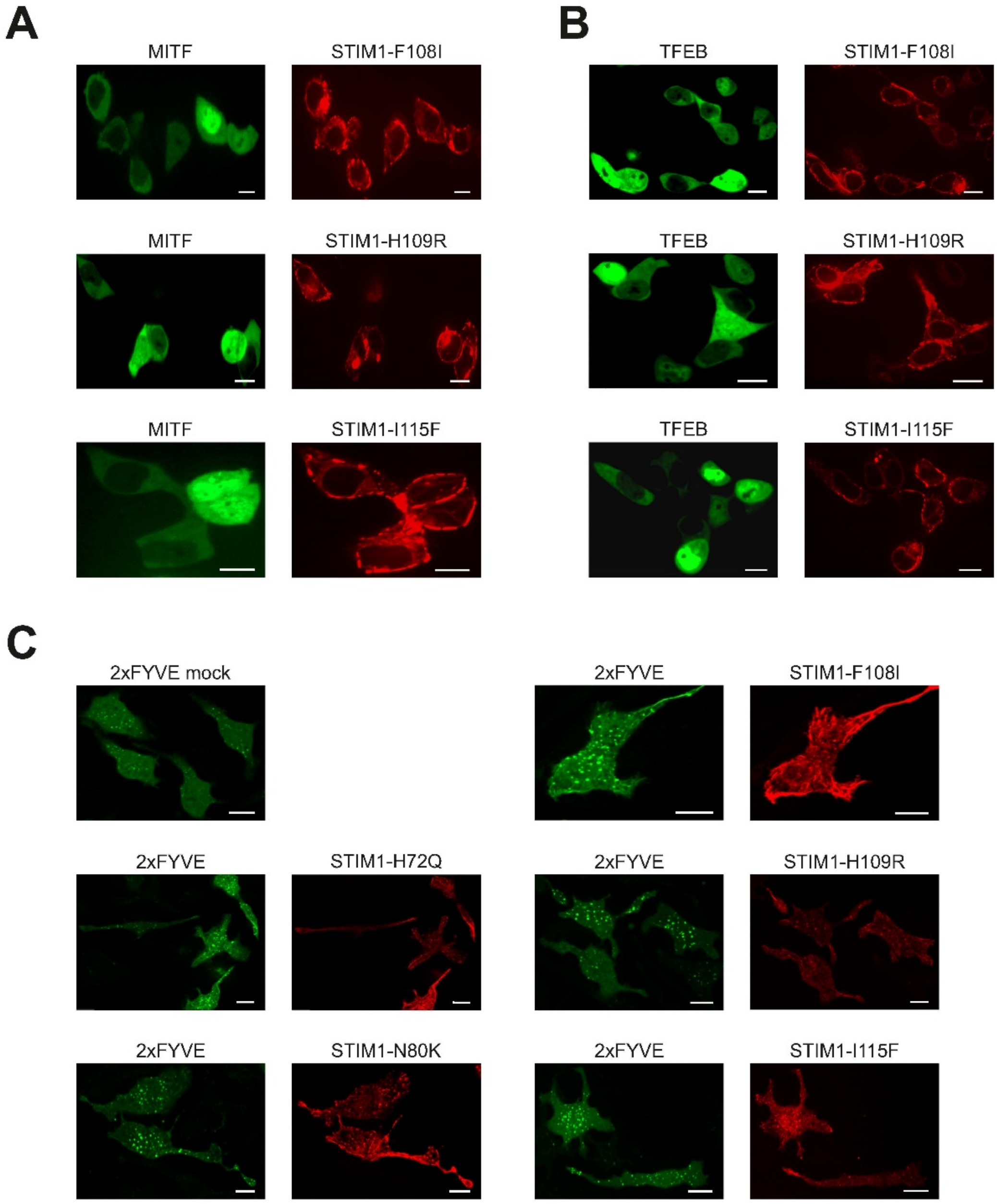

Supplementary Figure 2: Activation of autophagy and transcriptional pathways associated with disease-causing canonical and non-canonical STIM1 mutants. **(a-b)** Representative example images of cells co-expressing YFP-tagged STIM1-F108I, STIM1-H109R, STIM1-I115F and a) CFP-tagged MITF or b) CFP-tagged TFEB. Scale bar 10 µm. **(c)** Additional representative example images of cells co-overexpressing lysosomal marker 2xFYVE (mock) as well as STIM1-H72Q, STIM1-N80K, STIM1-F108I, STIM1-H109R, STIM1-I115F, respectively. Scale bar 10 µm.
